# Biodegradable Polylactide/TiO_2_ Composite Fiber Scaffolds with Superhydrophobic and Superadhesive Porous Surfaces for Water Immobilization, Antibacterial Performance, and Deodorization

**DOI:** 10.3390/polym11111860

**Published:** 2019-11-11

**Authors:** Xiaowen Wang, Dongchu Chen, Min Zhang, Huawen Hu

**Affiliations:** 1 School of Materials Science and Energy Engineering, Foshan University, Foshan, Guangdong 528000, China; wxwfky@126.com (X.W.); cdcever@163.com (D.C.); zhangmin@gic.ac.cn (M.Z.); 2 Guangdong Provincial Special Polymer Engineering Center for Building Materials, Guangdong 528000, China; 3 Guangdong Provincial Key Laboratory of New and Renewable Energy Research and Development, Guangzhou Institute of Energy Conversion, Chinese Academy of Sciences, Guangzhou 510640, China; 4 Guangdong Provincial Key Laboratory of Industrial Surfactant, Guangdong Research Institute of Petrochemical and Fine Chemical Engineering, Guangzhou 510640, China

**Keywords:** biodegradable polylactide, composite fiber mat, superhydrophobic, super-adhesive, multiple useful functions

## Abstract

In this short communication, TiO_2_-nanoparticle-functionalized biodegradable polylactide (PLA) nonwoven scaffolds with a superhydrophobic and superadhesive surface are reported regarding their water immobilization, antibacterial performance, and deodorization. With numerous regular oriented pores on their surface, the as-fabricated electrospun porous PLA/TiO_2_ composite fibers possessed diameters in the range from 5 µm down to 400 nm, and the lengths were even found to be up to the meters range. The PLA/TiO_2_ composite fiber surface was demonstrated to be both superhydrophobic and superadhesive. The size of the pores on the fiber surface was observed to have a length of 200 ± 100 nm and a width of 150 ± 50 nm using field-emission scanning electron microscopy and transmission electron microscopy. The powerful adhesive force of the PLA/TiO_2_ composite fibers toward water droplets was likely a result of van der Waals forces and accumulated negative pressure forces. Such a fascinating porous surface (functionalized with TiO_2_ nanoparticles) of the PLA/TiO_2_ composite fiber scaffold endowed it with multiple useful functions, including water immobilization, antibacterial performance, and deodorization.

## 1. Introduction

Controlling the wettability of solid surfaces is of abiding biological and technological significance for numerous processes in living organisms and considerable industrial applications [1,2,3,4,5,6,7,8,9,10,11,12,13]. Recently, the fabrication of superhydrophobic surfaces that can pin water droplets has aroused tremendous attention [8,9,10,11,12,13]. The microscopic mechanisms underlying the water droplet adhesion to the superhydrophobic surface have been extensively explored [4,5,6,8,9,13,14]. The ability to maintain the position of nearly spherical water droplets on a well-defined hydrophobic surface provides promise toward a wide range of applications, such as single molecular spectroscopy [13]; liquid transportation without loss, which facilitates the analysis of liquid samples in a trace quantity [9]; and painless wound sealing, which show high potential as a substitute for sutures or staples [14]. For the creation of this kind of functional surface, efforts are mainly directed to the fabrication of nanotip arrays on a wide range of substrates, such as flat silicon [13], poly(vinyl alcohol) films [12], a silanized nanotextured silicon wafer coated with hydroxyl-terminated polydimethylsiloxane [11], aligned polystyrene nanotubes [9,10], poly(methyl methacrylate)/amphiphilic polyurethane (PU)/fluorinated PU ternary blend [8], poly(glycerol-co-sebacate) acrylate [14], and polydopamine-functionalized TiO_2_ nanotube arrays incorporating SiO_2_ nanoparticles [15]. Many coating techniques can be used to construct functional surfaces, which are described in a recent review article [16]. However, little attention is paid to functional surfaces based on biodegradable and biocompatible polymers, which is expected to show more promising applications in medical therapies, waterproof sealants, and mesh grafts [14]. Because aliphatic polyesters, including polylactide (PLA) and polylactic acid, are readily available, non-toxic, renewable, biodegradable, sustainable, and biocompatible, they have been receiving considerable attention, and many potential applications have been explored, such as packaging [17,18], orthopedics [19], drug delivery [20,21], drug encapsulation [22], sutures [23], scaffolds [24,25], implants [26,27], antibacterial agents, and hemostatic applications [28].

Electrospinning technologies provide versatile routes to the fabrication of different biodegradable and biocompatible polymer-based fibers [29], which also makes it facile to endow the fiber material with hierarchical structures, functionalization, and intelligence through the wet spinning of the mixed solution with functional inorganic and organic additives [29]. As the most popular semiconductor photocatalyst, TiO_2_ has gained substantial interest as a functional additive for the fabrication of many kinds of functional composites over the last few decades due to its nontoxicity, biocompatibility, corrosion resistance, low cost, and visible-light-induced self-cleaning and antimicrobial performance [15,28,30,31]. In this article, we describe a straightforward and reliable approach for the fabrication of multifunctional electrospun (ES) biodegradable PLA/TiO_2_ nonwoven scaffolds. The PLA/TiO_2_ fiber surface was demonstrated to be porous and superhydrophobic, with a water contact angle (CA) of more than 150°. More importantly, it exhibited highly adhesive forces toward water droplets, which allowed the water droplets to be stably immobilized on the surface. The pinned water droplets did not roll and fall when the surface was vertical and inverted, respectively. Apart from the fascinating water immobilization performance, the as-spun PLA/TiO_2_ nanofiber mat also showed other useful functions, including antibacterial and deodorization performance, which therefore holds substantial promise for biomedical engineering applications. For example, the developed ES porous PLA/TiO_2_ composite fibers can be used to reduce bacterial infections through the inhibition of bacterial growth and adhesion. Specifically, during the crucial perioperative period, the bacterial adsorption and growth on the implants, equipped with the ES PLA/TiO_2_ fiber mat, can be suppressed. It also holds the potential to be used as a wound dressing for prohibiting the spread of odor and bacteria breeding. Furthermore, it shows promise toward the stabilization of droplets in microfluidic diagnostics.

## 2. Experimental

### 2.1. Materials

PLA (molecular weight of 1.0 × 10^6^ kg/mol, analytical reagent grade) was supplied by Natureworks Co. (Minnetonka, MN, USA). Chloroform and acetone of analytical reagent grade were obtained from Labscan Asia Co. Ltd. (Bangkok, Thailand), and Duksan pure chemicals Co. Ltd. (Ansan, Korea), respectively. Titanium (IV) isopropoxide (TIP, 98+%), hydrochloric acid (36.5%), and acetic acid (97%) were purchased from Sigma-Aldrich Chemical Co., Ltd (St. Louis, MO, USA). Polysorbate-80 (analytical reagent) was purchased from Sinopharm Chemical Reagent Co, Ltd. (Beijing, China). Sodium chloride (analytical reagent grade, mass fraction >99 %) was supplied by Tianjin Guangfu Chemical Reagent Co. Ltd. (Tianjin, China). All of the other reagents were of analytical purity and purchased from Sigma-Aldrich Chemical Co., Ltd (St. Louis, MO, USA). The materials were used without further purification unless otherwise stated.

### 2.2. Fabrication of the ES PLA/TiO_2_ fiber scaffold

In a typical procedure, 5 g of PLA was dissolved in a mixed solvent of chloroform (63 g) and acetone (32 g) at 150 °C under reflux for 2 h. Separately, a 10 wt% TiO_2_ sol was synthesized. Briefly, the 10 wt% TiO_2_ sol was prepared based on the following procedures: 75 mL TIP was added dropwise into acidic water (1.1 mL hydrochloric acid and 0.8 mL acetic acid) at 80 °C under vigorous stirring conditions. The mixtures were further stirred for 16 h to obtain the 10 wt% TiO_2_ sol. The PLA/TiO_2_ composite fiber mat was synthesized according to the following procedure: a mixture of 10 wt% TiO_2_/PLA was prepared by adding the as-synthesized TiO_2_ sol (5 g, 10 wt%) into the PLA solution (100 g, 5 wt%). The solution was placed in a syringe (10 mL) equipped with a cylindrical metal spinneret bearing an inner diameter of 0.8 mm and a wall thickness of 0.05 mm. The spinneret was connected to an electrode via an alligator clip. The electrode was connected to a high-voltage power supply and charged with a positive DC voltage up to 30 kV. The ES PLA/TiO_2_ fiber mats were collected from a grounded rotating drum at a target speed of 1.0 m/min. The spinning solution was delivered at a syringe pump speed of 0.02 mm/h, an applied voltage of 16 kV, a leakage current of 0.04 µA, a traverse speed of 20 cm/min, and a distance of 10 cm between the tip of the conical spinneret and the collector. The resulting ES PLA/TiO_2_ fiber scaffolds were immersed in ethanol for 30 min and dried for 24 h under vacuum to remove any solvent residues. The mass of the dried PLA/TiO_2_ fiber scaffolds was measured to be 4.03 g. The yield of the PLA/TiO_2_ composite fiber scaffold produced was thus calculated to be 73.3% according to Equation (1):

(1) $$Yield(\%)=\frac{m(PLA/TiO_{2})}{m(PLA)+m(TiO_{2})}\times 100$$

where m(PLA/TiO_2_) represents the mass of the final product generated, m(PLA) is the mass of the polymer PLA dissolved into the mixed solution that was prepared for electrospinning, and m(TiO_2_) denotes the mass of the TiO­_2_ nanoparticles dispersed in the mixed solution that was prepared for electrospinning.

### 2.3. Characterizations

A nanofiber electrospinning unit was purchased from the Kato Tech Co., Ltd. (Kyoto, Japan). The morphologies of the as-spun PLA/TiO_2_ fiber mat were investigated using field-emission scanning electron microscopy (FESEM, JSM–6335F at 3.0 kV, JEOL, Tokyo, Japan). Before the SEM observation, the sample surface was sputtered with a conductive gold coating under vacuum conditions. The lattice spacing was determined using high-resolution transmission electron microscopy (HRTEM, JEOL JEM 2010 operated at 200 kV, Tokyo, Japan). CAs were measured with a DataPhysics OCA20 CA system (Filderstadt, Germany) at ambient temperature according to the sessile method. For a typical CA measurement, water droplets with a quantitative volume of 4.0 µL were dispensed onto the surface of the PLA/TiO_2_ fiber mat. The average CAs were obtained by measuring at least five different positions of the same samples.

### 2.4. Antibacterial Activity Measurement

According to the ISO 27447:2009 standard “Test method for antibacterial activity of semiconducting photocatalytic materials,” the antibacterial performance of the prepared ES PLA/TiO_2_ scaffold was measured. Specifically, the bacterial solution with a concentration of ≈1.0 × 10^5^–1.0 × 10^6^ CFU was selected for the antibacterial evaluation. A given amount of the bacterial solution was added drop-by-drop onto the specimen, which was cultured for 24 h for the antibacterial evaluation. Separately, an eluent was prepared based on the following procedure: sodium chloride (8.5 g) was dissolved into deionized (DI) water (1000 mL), and then polysorbate-80 (a non-ionic surfactant, 2.0 g) was added. A portion of the mixture (20 mL) was added to a test tube and sterilized with an autoclave, producing the physiological saline solution (PSS) as the eluent that was kept in storage at 5 °C before use. A given amount of the prepared eluent PSS was employed to elute the specimen thoroughly, followed by counting of the viable organisms that existed in the eluted solution. The counting method was undertaken as follows: the eluted solution (1 mL) was fetched using a sterilized pipette and homogenized with the PSS (9 mL) in a test tube. A portion of the solution (1 mL) was withdrawn with another sterilized pipette and added to another test tube containing the PSS (9 mL). The process was repeated many times to obtain a series of dilutions. Afterwards, two portions of each dilution (1 mL each) were withdrawn into two parallel Petri dishes. Nutrient agar (16 mL) was then added to each Petri dish, which was allowed to stand for 15 min at room temperature. Upon the solidification of the agar medium, the Petri dishes were placed upside down and incubated for 40 h at 37 ± 1 °C. The colony numbers were finally counted in the Petri dishes, and the bacterial concentration of the eluted solution (*C*) was calculated based on Equation (2):

*C* = *N*_1_ × *T*, (2)

where *N*_1_ and *T* represent the average number of the colonies in the two Petri dishes and the dilution times, respectively.

The number of viable bacteria (*N*) on the specimen was estimated according to Equation (3):

*N* = *C* × *V*, (3)

where *V* is the volume (mL) of the PSS adopted for elution.

The specimens were cut from the ES PLA/TiO_2_ composite fiber mat, with the area of 1 × 10 cm^2^ for the antibacterial test, and three kinds of bacteria were adopted as the target to evaluate the broad-spectrum antibacterial activity, including *Staphylococcus aureus*, *Escherichia coli*, and *Candida albicans*. The visible lamp (with the ultraviolet light filtered, leaving the visible light across the wavelengths from 400 to 800 nm) was placed in front of the incubator door as the light source with the illumination intensity measured as 500–600 lux using a digital light meter.

### 2.5. Deodorization Performance Evaluation

According to the ISO 22197-4:2013 standard “Test method for air-purification performance of semiconducting photocatalytic materials,” the illumination intensity of artificial visible light (with the wavelength range of 400–800 nm) was measured to be 500–600 lux using a digital light meter. The ISO 22197-4:2013 standard was established for the evaluation of the deodorization performance of different types of materials, including woven and nonwoven fabrics, plastic, paper materials, etc. In this study, the deodorization test was carried out in an airtight laboratory chamber with a volume of 1 m^3^. A piece of the ES PLA/TiO_2_ sample (with the area of 1 m^2^) was adhered to each inner side of the test chamber, and the visible lamp was fixed in the center of the chamber. The intensity of the visible light illuminated on each side was measured to be 500–600 lux with a digital light meter. Two typical pollutants were employed for the evaluation of the deodorization performance, including ammonia and formaldehyde, whose concentrations were probed using an AR8500 ammonia gas detector (Shenzhen Graigar Technology Co., Ltd., Shenzhen, China) and a USA ESC Z-300XP formaldehyde detector (Environmental Sensors Co., Florida, USA), respectively. A certain amount of pollutants was injected into the chamber. Next, the concentration of the pollutant in the chamber was examined. The deodorization test was initially performed under dark conditions for 48 h to ensure an equilibrium state, and the equilibrium pollutant concentrations were recorded. The visible light irradiation was subsequently applied to the equilibrium system, and the photocatalytic reaction over the ES PLA/TiO_2_ specimen was sustained for 2 h. The pollutant removal rate was calculated based on the difference between the pollutant concentrations in the equilibrium chamber under dark conditions and in the chamber that was subject to an additional 2 h of visible light irradiation.

## 3. Results and Discussion

**Figure 1:**
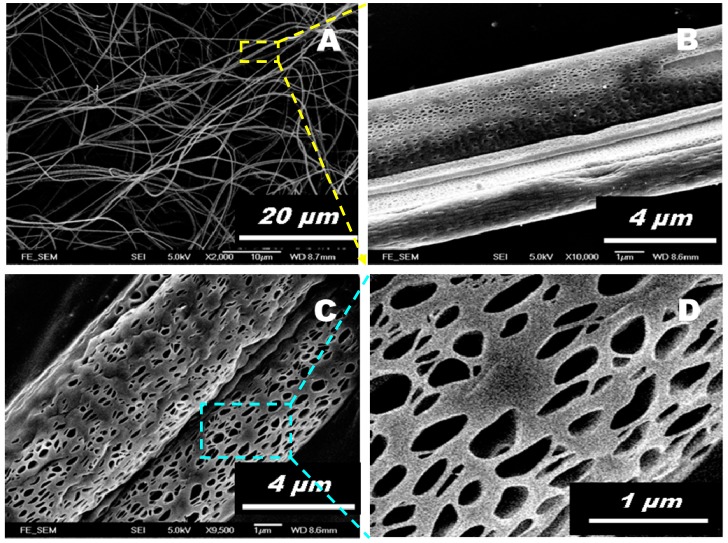
FESEM images of the prepared ES PLA/TiO_2_ fibers at different magnification scales. Note that the images (**B**,**D**) are magnified from the locations indicated in images (**A**,**C**), respectively.

**Figure 2:**
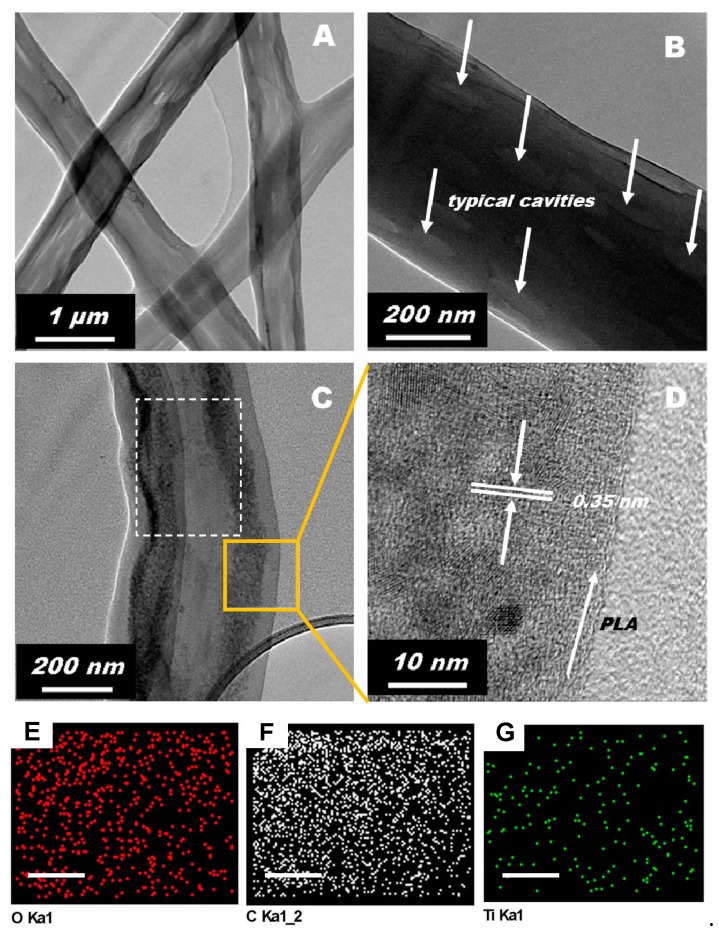
TEM images of the prepared typical ES PLA/TiO_2_ fibers. (**A**) Low-magnification TEM image of ES PLA/TiO_2_ fibers. (**B**) Magnified TEM image of an ES PLA/TiO_2_ fiber captured for the observation of typical cavities. (**C**) Magnified TEM image of an ES PLA/TiO_2_ fiber captured for the observation of TiO_2_ nanoparticles. (**D**) High-resolution TEM image zoomed in from the marked area in (**C**) for the identification of the lattice fringe of TiO_2_ nanocrystallites. (**E**–**G**) The O, C, and Ti element mapping images of the location indicated in image (**C**) with a dashed rectangle. The scale bars in all the mapping images (**E**–**G**) are 100 nm.

The surface morphology of the meso-scaled ES PLA/TiO_2_ fiber mat was demonstrated using SEM and TEM (Figure 1 and Figure 2), which confirmed its successful fabrication. The as-fabricated ES PLA/TiO_2_ fibers were morphologically uniform, with numerous elongated holes distributed over the fiber surface, as shown in Figure 1A–D. The ES PLA/TiO_2_ composite fibers exhibited diameters ranging from 400 nm to 5 µm and the lengths were even found to be up to the meters range. Typical cavities [32] observed on the surface of the cylinder fibers are shown in Figure 1B–D and Figure 2A,B, with the length and width measured to be 200 ± 100 nm and 150 ± 50 nm, respectively. The formation of such regular pores (elongated along the fiber axis) on the as-spun PLA/TiO_2_ fiber surface resulted from a rapid phase separation during the electrospinning process as induced by the volatile solvent evaporation and the subsequent fast solidification. The regular phase morphology was generated as a consequence of the rapid phase separation during the electrospinning course, and the solvent-rich regions were transformed into cavities.

A low-magnification TEM image of the ES PLA/TiO_2_ fibers was presented in Figure 2A. The cavities on the surface of the typical fibers are marked in Figure 2B. The TiO_2_ species incorporated in the PLA fiber matrix are identified in Figure 2C, and the marked area in Figure 2C was further zoomed in to yield the high-resolution TEM image, as provided in Figure 2D. As is marked as parallel white lines in Figure 2D, the adjacent lattice fringe spacing was measured to be 0.35 nm, corresponding to the (101) crystallographic plane of anatase TiO_2_ [33]. TiO_2_ was crystallized to form crystallites with a dimension of 5–10 nm, similar to a previous report [28]. The distribution of the TiO_2_ nanoparticles was also examined over a large domain of the ES PLA/TiO_2_ composite fiber (marked with a dashed rectangle in Figure 2C) using the EDX elemental mapping technique (Figure 2E–G), and a uniform Ti element distribution could be observed (Figure 2G), revealing that TiO_2_ nanoparticles were homogeneously dispersed in the PLA fiber matrix. The polymer fiber alignment was also observed and marked, in stark contrast to the whiter background of the carbon grid (Figure 2D).

**Figure 3:**
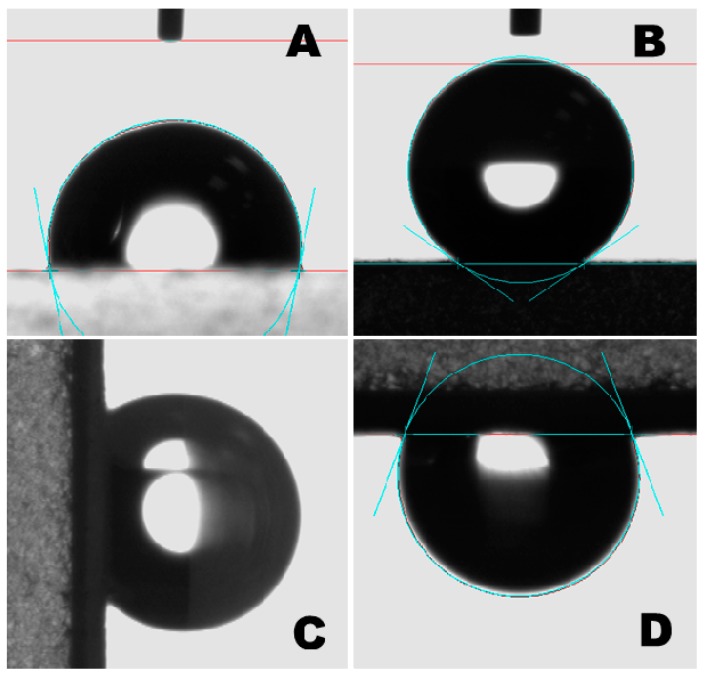
Optical images of a 4 µL water droplet positioned on different substrates. (**A**) The water droplet on a bulk PLA film/glass wafer substrate; the water CA for this drop was 101°. (**B**–**D**) Water droplets on an ES PLA/TiO_2_ fiber mat/glass wafer substrate at different tilted angles: (**B**) the water CA was measured as 150° when the tilted angle was 0°; (**C**) the water droplet shape was deformed by its gravity to some extent when the tilted angle was 90°, but the droplet did not roll or slide along the substrate surface; and (**D**) the water droplet was suspended upside-down via manually inverting the substrate, i.e., with the tilted angle of 180°. All of these results demonstrated that the ES PLA/TiO_2_ fiber mat-modified surface exhibited a highly adhesive force on water droplets, which could be attributed to the strong van der Waals forces between the water droplets and contact surface of the composite fiber mat, and to the accumulated negative pressure forces as generated by the increment in the volume of air pockets being isolated by the covered droplets.

**Figure 4:**
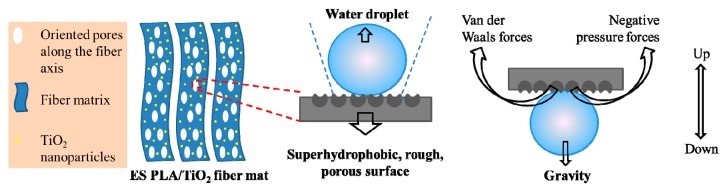
Scheme for showing the water immobilization on the superhydrophobic and superadhesive porous surface of the as-spun PLA/TiO_2_ fiber.

Bulk PLA was a hydrophobic material with a water CA of around 101°, as demonstrated in Figure 3A. The ES PLA/TiO_2_ fibers, however, showed superhydrophobicity with a CA of more than 150° (Figure 3B). Interestingly, the as-spun nonwoven scaffold demonstrated the ability to adhere to water droplets, as proven by perpendicularly tilting and inverting the positions of the surfaces with the water droplets, as shown in Figure 3C,D, respectively. The inversion of the surface dispersed with water droplets allowed for the easy estimation of the adhesive force between the water droplet and the ES PLA/TiO_2_ fiber substrate (Figure 3D). The adhesion force was more significant than the gravity force of the water droplets by considering that the adhesive force was strong enough to pin the water droplet. Such PLA/TiO_2_ fibers, therefore, had a much higher adhesive force than that of the aligned polystyrene nanotube films [8] with a maximum of 59.8 µN. The powerful adhesive force was likely due to van der Waals forces and the accumulated negative pressure forces of as-fabricated PLA/TiO_2_ fibers, as schematically displayed in Figure 4.

According to the ISO 27447:2009 standard, we further investigated the antibacterial performance of this attractive ES PLA/TiO_2_ fiber mat. Three typical kinds of microorganisms were tested, and the quantitative results of the antibacterial evaluations of the ES PLA/TiO_2_ specimen under the visible light are summarized in Table 1. After 24 h of visible light irradiation, the average number of the viable *Staphylococcus aureus* cells on the ES PLA/TiO_2_ specimen was reduced from 1.6 × 10^6^ to less than 20 CFU/piece, thus indicating that the antibacterial rate (*R*) was higher than 99% (Table 1). Under the same antibacterial processing conditions as that for *Staphylococcus aureus*, the antibacterial rates of the ES PLA/TiO_2_ specimen were measured to be higher than 99% and 93.3% for *Escherichia coli* and *Candida albicans*, respectively. Conversely, the average number of the viable bacteria cells on the standard control test specimen in the absence of the ES PLA/TiO_2_ fibers was dramatically increased after the 24 h of visible light irradiation, likely because bacterial proliferation readily proceeded without the inhibition effect from the destructive reactive oxygen species generated by the photocatalytic reactions over the PLA/TiO_2_ fibers. As a result, the as-prepared PLA/TiO_2_ fibers can find promising antimicrobial applications by considering its high antibacterial efficiency, as well as its robust and portable merits that cannot be obtained in the traditional powdery samples [34,35,36,37,38,39,40,41,42,43,44,45,46].

*R*_1_ = ((*N*_0_ – *N*_24h_)/*N*_0_) × 100 (4)

According to the ISO 22197-4:2013 standard, the deodorization performance of the prepared ES PLA/TiO_2_ fiber mat was evaluated. The 500–600 lux illumination intensity of the artificial visible light was adopted for the present test on deodorization based on the visible-light-driven photocatalytic reactions over the prepared ES PLA/TiO_2_ fiber mat specimen. The deodorization test was carried out in a 1-m^3^ laboratory chamber. The ES PLA/TiO_2_ fiber mat specimen adhered to the four sides of the test chamber. Two typical kinds of pollutants were used for testing, including ammonia and formaldehyde. The deodorization results of the ES PLA/TiO_2_ specimen under visible light are provided in Table 2. After 2 h of visible light irradiation, the concentrations of ammonia and formaldehyde were reduced by 76.2% and 63.6%, respectively. These results demonstrated that the as-prepared PLA/TiO_2_ composite fibers also possessed a high deodorization efficiency, in addition to the impressive antibacterial performance and water droplet immobilization functions.

*R*_2_ = ((*C*_0_ − *C*_2h_)/*C*_0_) × 100 (5)

## 4. Conclusions

A multifunctional, biodegradable PLA/TiO_2_ nonwoven fiber scaffold was presented, with many useful findings disclosed. First, the surface of the PLA/TiO_2_ composite fiber was demonstrated with regular pores oriented along the fiber axis. Second, such a porous surface was also verified to be superhydrophobic (with a water CA of more than 150°). Third, the porous surface was also shown to be superadhesive toward water droplets (as evidenced by the water droplets still attached to the surface when it was tilted perpendicularly and even inverted). The powerful adhesive force was likely due to van der Waals forces and the accumulated negative pressure forces of the as-spun PLA/TiO_2_ fibers. Fourth, due to the unique structural feature of the PLA/TiO_2_ composite nanofibers, they also exhibited highly efficient antibacterial performance toward three kinds of bacteria, namely *Staphylococcus aureus*, *Escherichia coli*, and *Candida albicans*, under visible light irradiation, and the antibacterial rates were estimated to be higher than 93% for all the bacteria tested. Fifth, effective deodorization performance of the ES PLA/TiO_2_ fibers toward ammonia and formaldehyde was also demonstrated. Taken together, the multiple functions of the as-spun PLA/TiO_2_ fiber mat will bring many benefits to real-world biomedical and bioengineering applications.

## Figures and Tables

**Table 1:** Test results of the antibacterial performance of the ES PLA/TiO_2_ fiber mat.

Test Microorganism	*N*_0_^a^ (CFU/piece)	*N*_24h_^b^ for the Control Specimen (CFU/piece)	*N*_24h_^c^ for the ES PLA/TiO_2_ Specimen (CFU/piece)	*R*_1_^d^ (%)
*Staphylococcus aureus*	2.4 × 10^5^	1.6 × 10^6^	< 20	> 99
*Escherichia coli*	2.1 × 10^5^	2.5 × 10^5^	< 20	> 99
*Candida albicans*	2.7 × 10^5^	3.9 × 10^5^	1.8 × 10^4^	93.3

Note: ^a^ The average number of viable bacteria cells before inoculation with specimens. ^b^ The average number of the viable bacteria cells on the standard control test specimen after incubation under visible light irradiation for 24 h. ^c^ The average number of viable bacteria cells on the PLA/TiO_2_ specimen after incubation under visible light irradiation for 24 h. ^d^ Antibacterial rate calculated for the PLA/TiO_2_ specimen according to Equation (4).

**Table 2:** Test results of deodorization performance of the ES PLA/TiO_2_ specimen.

Test Pollutants	Concentration of Pollutants (mg/m^3^)		*R*_2_^c^ (%)
	*C*_0_^a^	*C*_2h_^b^	
Ammonia	1.01	0.24	76.2
Formaldehyde	1.07	0.39	63.6

Note: ^a^ The equilibrium concentration of the pollutant in the laboratory chamber equipped with the PLA/TiO_2_ specimen under dark conditions for 48 h before the visible light was turned on. ^b^ The residual concentration of the pollutant in the laboratory chamber equipped with the PLA/TiO_2_ specimen after 2 h of visible-light irradiation. ^c^ Removal rate, which was calculated according to Equation (5).
